# Supplementary material for: HIVprotI: an integrated web based platform for prediction and design of HIV proteins inhibitors
Source: J Cheminform. 2018 Mar 9;10:12. doi: 10.1186/s13321-018-0266-y (PMC5845081; doi:10.1186/s13321-018-0266-y)
Supplement: Supplementary file 2 — Additional file 2. Source code of HIVProtI web server. [file 13321_2018_266_MOESM2_ESM.zip › HIVprotI_Source-code/hivprotI/weka-3-6-6/doc/weka/gui/GenericArrayEditor.html]

GenericArrayEditor


---


|  |  |  |
| --- | --- | --- |
| **Overview** \| **Package** \| **Class** \| **Tree** \| **Deprecated** \| **Index** \| **Help** \| **Weka's home** | |  |
| **PREV CLASS**   **NEXT CLASS** | **FRAMES**    **NO FRAMES**     **All Classes** |  |
| SUMMARY: NESTED \| FIELD \| CONSTR \| METHOD | DETAIL: FIELD \| CONSTR \| METHOD |  |


---


## weka.gui Class GenericArrayEditor

```
java.lang.Object
  java.awt.Component
      java.awt.Container
          javax.swing.JComponent
              javax.swing.JPanel
                  weka.gui.GenericArrayEditor
```

**All Implemented Interfaces:**: java.awt.image.ImageObserver, java.awt.MenuContainer, java.beans.PropertyEditor, java.io.Serializable, javax.accessibility.Accessible

---

``` public class GenericArrayEditor extends javax.swing.JPanel implements java.beans.PropertyEditor ```

A PropertyEditor for arrays of objects that themselves have
property editors.

**Version:**
:   $Revision: 7651 $

**Author:**
:   Len Trigg (trigg@cs.waikato.ac.nz)

**See Also:**: Serialized Form

---

**Nested Class Summary**

**Nested classes/interfaces inherited from class javax.swing.JComponent**

`javax.swing.JComponent.AccessibleJComponent`

**Nested classes/interfaces inherited from class java.awt.Component**

`java.awt.Component.BaselineResizeBehavior`


**Field Summary**

**Fields inherited from class javax.swing.JComponent**

`TOOL_TIP_TEXT_KEY, UNDEFINED_CONDITION, WHEN_ANCESTOR_OF_FOCUSED_COMPONENT, WHEN_FOCUSED, WHEN_IN_FOCUSED_WINDOW`

**Fields inherited from class java.awt.Component**

`BOTTOM_ALIGNMENT, CENTER_ALIGNMENT, LEFT_ALIGNMENT, RIGHT_ALIGNMENT, TOP_ALIGNMENT`

**Fields inherited from interface java.awt.image.ImageObserver**

`ABORT, ALLBITS, ERROR, FRAMEBITS, HEIGHT, PROPERTIES, SOMEBITS, WIDTH`


**Constructor Summary**

`GenericArrayEditor()`
  
          Sets up the array editor.


| **Method Summary** | |
| --- | --- |
| `void` | `addPropertyChangeListener(java.beans.PropertyChangeListener l)`             Adds a PropertyChangeListener who will be notified of value changes. |
| `java.lang.String` | `getAsText()`             Returns null as we don't support getting/setting values as text. |
| `java.awt.Component` | `getCustomEditor()`             Returns the array editing component. |
| `java.lang.String` | `getJavaInitializationString()`             Supposedly returns an initialization string to create a classifier identical to the current one, including it's state, but this doesn't appear possible given that the initialization string isn't supposed to contain multiple statements. |
| `java.lang.String[]` | `getTags()`             Returns null as we don't support getting values as tags. |
| `java.lang.Object` | `getValue()`             Gets the current object array. |
| `boolean` | `isPaintable()`             Returns true to indicate that we can paint a representation of the string array. |
| `static void` | `main(java.lang.String[] args)`             Tests out the array editor from the command line. |
| `static java.lang.Object` | `makeCopy(java.lang.Object source)`             Makes a copy of an object using serialization. |
| `void` | `paintValue(java.awt.Graphics gfx, java.awt.Rectangle box)`             Paints a representation of the current classifier. |
| `void` | `removePropertyChangeListener(java.beans.PropertyChangeListener l)`             Removes a PropertyChangeListener. |
| `void` | `setAsText(java.lang.String text)`             Returns null as we don't support getting/setting values as text. |
| `void` | `setValue(java.lang.Object o)`             Sets the current object array. |
| `boolean` | `supportsCustomEditor()`             Returns true because we do support a custom editor. |

**Methods inherited from class javax.swing.JPanel**

`getAccessibleContext, getUI, getUIClassID, setUI, updateUI`

**Methods inherited from class javax.swing.JComponent**

`addAncestorListener, addNotify, addVetoableChangeListener, computeVisibleRect, contains, createToolTip, disable, enable, firePropertyChange, firePropertyChange, firePropertyChange, getActionForKeyStroke, getActionMap, getAlignmentX, getAlignmentY, getAncestorListeners, getAutoscrolls, getBaseline, getBaselineResizeBehavior, getBorder, getBounds, getClientProperty, getComponentPopupMenu, getConditionForKeyStroke, getDebugGraphicsOptions, getDefaultLocale, getFontMetrics, getGraphics, getHeight, getInheritsPopupMenu, getInputMap, getInputMap, getInputVerifier, getInsets, getInsets, getListeners, getLocation, getMaximumSize, getMinimumSize, getNextFocusableComponent, getPopupLocation, getPreferredSize, getRegisteredKeyStrokes, getRootPane, getSize, getToolTipLocation, getToolTipText, getToolTipText, getTopLevelAncestor, getTransferHandler, getVerifyInputWhenFocusTarget, getVetoableChangeListeners, getVisibleRect, getWidth, getX, getY, grabFocus, isDoubleBuffered, isLightweightComponent, isManagingFocus, isOpaque, isOptimizedDrawingEnabled, isPaintingForPrint, isPaintingTile, isRequestFocusEnabled, isValidateRoot, paint, paintImmediately, paintImmediately, print, printAll, putClientProperty, registerKeyboardAction, registerKeyboardAction, removeAncestorListener, removeNotify, removeVetoableChangeListener, repaint, repaint, requestDefaultFocus, requestFocus, requestFocus, requestFocusInWindow, resetKeyboardActions, reshape, revalidate, scrollRectToVisible, setActionMap, setAlignmentX, setAlignmentY, setAutoscrolls, setBackground, setBorder, setComponentPopupMenu, setDebugGraphicsOptions, setDefaultLocale, setDoubleBuffered, setEnabled, setFocusTraversalKeys, setFont, setForeground, setInheritsPopupMenu, setInputMap, setInputVerifier, setMaximumSize, setMinimumSize, setNextFocusableComponent, setOpaque, setPreferredSize, setRequestFocusEnabled, setToolTipText, setTransferHandler, setVerifyInputWhenFocusTarget, setVisible, unregisterKeyboardAction, update`

**Methods inherited from class java.awt.Container**

`add, add, add, add, add, addContainerListener, addPropertyChangeListener, applyComponentOrientation, areFocusTraversalKeysSet, countComponents, deliverEvent, doLayout, findComponentAt, findComponentAt, getComponent, getComponentAt, getComponentAt, getComponentCount, getComponents, getComponentZOrder, getContainerListeners, getFocusTraversalKeys, getFocusTraversalPolicy, getLayout, getMousePosition, insets, invalidate, isAncestorOf, isFocusCycleRoot, isFocusCycleRoot, isFocusTraversalPolicyProvider, isFocusTraversalPolicySet, layout, list, list, locate, minimumSize, paintComponents, preferredSize, printComponents, remove, remove, removeAll, removeContainerListener, setComponentZOrder, setFocusCycleRoot, setFocusTraversalPolicy, setFocusTraversalPolicyProvider, setLayout, transferFocusBackward, transferFocusDownCycle, validate`

**Methods inherited from class java.awt.Component**

`action, add, addComponentListener, addFocusListener, addHierarchyBoundsListener, addHierarchyListener, addInputMethodListener, addKeyListener, addMouseListener, addMouseMotionListener, addMouseWheelListener, bounds, checkImage, checkImage, contains, createImage, createImage, createVolatileImage, createVolatileImage, dispatchEvent, enable, enableInputMethods, firePropertyChange, firePropertyChange, firePropertyChange, firePropertyChange, firePropertyChange, getBackground, getBounds, getColorModel, getComponentListeners, getComponentOrientation, getCursor, getDropTarget, getFocusCycleRootAncestor, getFocusListeners, getFocusTraversalKeysEnabled, getFont, getForeground, getGraphicsConfiguration, getHierarchyBoundsListeners, getHierarchyListeners, getIgnoreRepaint, getInputContext, getInputMethodListeners, getInputMethodRequests, getKeyListeners, getLocale, getLocation, getLocationOnScreen, getMouseListeners, getMouseMotionListeners, getMousePosition, getMouseWheelListeners, getName, getParent, getPeer, getPropertyChangeListeners, getPropertyChangeListeners, getSize, getToolkit, getTreeLock, gotFocus, handleEvent, hasFocus, hide, imageUpdate, inside, isBackgroundSet, isCursorSet, isDisplayable, isEnabled, isFocusable, isFocusOwner, isFocusTraversable, isFontSet, isForegroundSet, isLightweight, isMaximumSizeSet, isMinimumSizeSet, isPreferredSizeSet, isShowing, isValid, isVisible, keyDown, keyUp, list, list, list, location, lostFocus, mouseDown, mouseDrag, mouseEnter, mouseExit, mouseMove, mouseUp, move, nextFocus, paintAll, postEvent, prepareImage, prepareImage, remove, removeComponentListener, removeFocusListener, removeHierarchyBoundsListener, removeHierarchyListener, removeInputMethodListener, removeKeyListener, removeMouseListener, removeMouseMotionListener, removeMouseWheelListener, removePropertyChangeListener, repaint, repaint, repaint, resize, resize, setBounds, setBounds, setComponentOrientation, setCursor, setDropTarget, setFocusable, setFocusTraversalKeysEnabled, setIgnoreRepaint, setLocale, setLocation, setLocation, setName, setSize, setSize, show, show, size, toString, transferFocus, transferFocusUpCycle`

**Methods inherited from class java.lang.Object**

`equals, getClass, hashCode, notify, notifyAll, wait, wait, wait`

**Constructor Detail**

### GenericArrayEditor

```
public GenericArrayEditor()
```

:   Sets up the array editor.


**Method Detail**

### setValue

```
public void setValue(java.lang.Object o)
```

:   Sets the current object array.

    :   **Specified by:**: `setValue` in interface `java.beans.PropertyEditor`
    :   **Parameters:**: `o` - an object that must be an array.

---


### getValue

```
public java.lang.Object getValue()
```

:   Gets the current object array.

    :   **Specified by:**: `getValue` in interface `java.beans.PropertyEditor`
    :   **Returns:**: the current object array

---


### getJavaInitializationString

```
public java.lang.String getJavaInitializationString()
```

:   Supposedly returns an initialization string to create a classifier
    identical to the current one, including it's state, but this doesn't
    appear possible given that the initialization string isn't supposed to
    contain multiple statements.

    :   **Specified by:**: `getJavaInitializationString` in interface `java.beans.PropertyEditor`
    :   **Returns:**: the java source code initialisation string

---


### isPaintable

```
public boolean isPaintable()
```

:   Returns true to indicate that we can paint a representation of the
    string array.

    :   **Specified by:**: `isPaintable` in interface `java.beans.PropertyEditor`
    :   **Returns:**: true

---


### paintValue

```
public void paintValue(java.awt.Graphics gfx,
                       java.awt.Rectangle box)
```

:   Paints a representation of the current classifier.

    :   **Specified by:**: `paintValue` in interface `java.beans.PropertyEditor`
    :   **Parameters:**: `gfx` - the graphics context to use: `box` - the area we are allowed to paint into

---


### getAsText

```
public java.lang.String getAsText()
```

:   Returns null as we don't support getting/setting values as text.

    :   **Specified by:**: `getAsText` in interface `java.beans.PropertyEditor`
    :   **Returns:**: null

---


### setAsText

```
public void setAsText(java.lang.String text)
```

:   Returns null as we don't support getting/setting values as text.

    :   **Specified by:**: `setAsText` in interface `java.beans.PropertyEditor`
    :   **Parameters:**: `text` - the text value **Throws:**: `java.lang.IllegalArgumentException` - as we don't support getting/setting values as text.

---


### getTags

```
public java.lang.String[] getTags()
```

:   Returns null as we don't support getting values as tags.

    :   **Specified by:**: `getTags` in interface `java.beans.PropertyEditor`
    :   **Returns:**: null

---


### supportsCustomEditor

```
public boolean supportsCustomEditor()
```

:   Returns true because we do support a custom editor.

    :   **Specified by:**: `supportsCustomEditor` in interface `java.beans.PropertyEditor`
    :   **Returns:**: true

---


### getCustomEditor

```
public java.awt.Component getCustomEditor()
```

:   Returns the array editing component.

    :   **Specified by:**: `getCustomEditor` in interface `java.beans.PropertyEditor`
    :   **Returns:**: a value of type 'java.awt.Component'

---


### addPropertyChangeListener

```
public void addPropertyChangeListener(java.beans.PropertyChangeListener l)
```

:   Adds a PropertyChangeListener who will be notified of value changes.

    :   **Specified by:**: `addPropertyChangeListener` in interface `java.beans.PropertyEditor` **Overrides:**: `addPropertyChangeListener` in class `java.awt.Container`
    :   **Parameters:**: `l` - a value of type 'PropertyChangeListener'

---


### removePropertyChangeListener

```
public void removePropertyChangeListener(java.beans.PropertyChangeListener l)
```

:   Removes a PropertyChangeListener.

    :   **Specified by:**: `removePropertyChangeListener` in interface `java.beans.PropertyEditor` **Overrides:**: `removePropertyChangeListener` in class `java.awt.Component`
    :   **Parameters:**: `l` - a value of type 'PropertyChangeListener'

---


### makeCopy

```
public static java.lang.Object makeCopy(java.lang.Object source)
```

:   Makes a copy of an object using serialization.

    :   **Parameters:**: `source` - the object to copy **Returns:**: a copy of the source object, null if copying fails

---


### main

```
public static void main(java.lang.String[] args)
```

:   Tests out the array editor from the command line.

    :   **Parameters:**: `args` - ignored


---


|  |  |  |
| --- | --- | --- |
| **Overview** \| **Package** \| **Class** \| **Tree** \| **Deprecated** \| **Index** \| **Help** \| **Weka's home** | |  |
| **PREV CLASS**   **NEXT CLASS** | **FRAMES**    **NO FRAMES**     **All Classes** |  |
| SUMMARY: NESTED \| FIELD \| CONSTR \| METHOD | DETAIL: FIELD \| CONSTR \| METHOD |  |


---
